# Identifying the poor for premium exemption: a critical step towards universal health coverage in Sub-Saharan Africa

**DOI:** 10.1186/s41256-016-0023-6

**Published:** 2017-01-09

**Authors:** Chukwuemeka A. Umeh

**Affiliations:** Department of Global Health, Boston University School of Public Health, Boston, MA USA

**Keywords:** Universal Health Coverage, Extreme Poverty, Means Testing, Health Insurance Premium, Catastrophic Expenditure

## Abstract

Premium exemption for the poor is a critical step towards achieving universal health coverage in sub-Saharan Africa due to the large proportion of the population living in extreme poverty who cannot pay premium. However, identifying the poor for premium exemption has been a big challenge for SSA countries. This paper is a succinct review of four methods available for identifying the poor, outlining the ideal conditions under which each of the methods should be used and the drawbacks associated with using each of the methods.

Universal health coverage (UHC) means that all people have access to needed promotive, preventive, curative, rehabilitative, and palliative health services, without suffering financial hardship due to the use of these health services [1]. UHC is built on a health financing system that encourages risk pooling and pre-payment contributions in order to avoid catastrophic expenditure due to out of pocket payment at the point of health service delivery [1].

Many sub-Saharan Africa (SSA) countries have made little progress towards UHC due to several factors one of which is that they have a large proportion of the population living in extreme poverty (below $1.25/day), who are unable to pay health insurance premiums [2]. These poor people are also further impoverished by out of pocket payments. Therefore premium exemption for the poor and vulnerable population is critical in achieving UHC.

However, one critical challenge in covering the poor in SSA is in identifying the poor. Although there are premium exemptions for the poor in some SSA countries such as Ghana and Tanzania, most of the exemptions are not well implemented because of difficulty in identifying the poor [3, 4]. In order to help SSA countries deal with the challenge of identifying the poor, I succinctly outlined four main methods of identifying the poor, the ideal conditions for using the different methods and the drawbacks in each of the methods that health insurance schemes in SSA should be aware of.

## Methods of identifying the poor

Based on literature review, the four main ways of identifying the poor which SSA countries can use are shown in Table 1. They include: means testing (identifying the poor using self-reported income or expenditure), proxy means testing (classifying socio-economic status based on ownership of assets and access to services), geographical targeting (classifying people based on where they live e.g., urban slums as poor) and participatory wealth ranking (community representatives rank households into socio-economic categories based on poverty indicators that the community decides) [5, 6].

**Table 1:** Methods of identifying the poor

Method	Ideal condition to use in sub-Saharan Africa	Drawback	Error of exclusion^a^
Means testing (MT)	None	Very expensive	No error of exclusion
Proximal means testing (PMT)	Low poverty incidence urban areas	Expensive, measures relative poverty	Possibility of significant error of exclusion
Geographic targeting (GT)	High poverty incidence areas (both urban and rural)	GT could lead to the non-poor who live in poor neighborhoods being exempted from premium	No error of exclusion
Participatory wealth ranking (PWR)	Low poverty incidence rural communities	Measures relative poverty, cannot be used where community ties are weak	Possibility of significant error of exclusion

^a^Error of exclusion - not identifying the poor as poor

**Means testing (MT)** is the most effective means of identifying the poor in both urban and rural areas because it correctly identifies the poor as poor [5]. However, the major drawback is that it is expensive because it involves collecting detailed data through a household survey. This is in addition to other challenges such as the difficulty of assigning monetary value to food which local farmers harvested from their farms, recall bias for expenditures etc. [5] So means testing might not be an appropriate method to use in most SSA countries because of the cost and logistics involved in the process.

**Proximal means testing (PMT)** is less expensive than MT and has been used in many social programs to identify the poor. PMT is ideal for relatively low poverty incidence urban areas [5]. However, the major drawback is that it has been associated with high exclusion error, with chances of excluding the poor ranging from 26 to 84% [5, 7, 8].

**Geographical targeting (GT)** is most appropriate in high poverty incidence areas (both urban and rural).^5^ However, one of the drawback is that GT could lead to the poor who are living in non-poor neighborhoods being excluded from premium exemption while the non-poor living in poor neighborhoods receive the premium exemption that they do not need [5].

**Participatory wealth ranking (PWR)** is very appropriate for relatively low poverty incidence rural communities [5]. It is a fast and less-resource intensive way of identifying the poor in rural communities [5, 9]. The challenge with the PWR is that it might be difficult to use in urban areas where the community ties are weak and people might not know each other very well [9]. Another challenge with PWR is that it measures relative poverty. So someone who might be seen as poor in one community might not be identified as poor in another community depending on the average level of wealth in the different communities.

## Conclusion

In summary, identifying the poor in SSA is a critical step towards UHC. However, this is only relevant in countries that have an insurance system that provides premium exemption for the poor. In some SSA countries like Nigeria, there is no premium exemption for the poor [10]. So providing premium exemptions alongside identifying the poor are important steps towards UHC in SSA due to the large proportion of people living in extreme poverty who cannot afford to pay premiums [2].

However, the different methods for identifying the poor have drawbacks and challenges such as high cost (MT and PMT), errors of inclusion, that is identifying the non-poor as poor (MT, PMT, GT, and PWR), and errors of exclusion, that is not identifying the poor as poor (PMT, PWR). If a country wants to ensure no poor person is excluded in a particular location, then MT or GT which have low or no error of exclusion will be most appropriate. To reduce inclusion error, countries can combine more than one method in a specific location such as first identifying the poor using PWR or GT and then using PMT to screen those identified. Although this will drive up cost of identifying the poor, in the long run it might save cost if there is high inclusion error.

So no one method of assessing the poor is ideal in all settings. It is therefore left to countries in SSA to use the method(s) that will be most appropriate for specific locations and circumstances while noting the possible drawbacks from the method(s).
